# Production and Characterization of k-Carrageenan Films Incorporating *Cymbopogon winterianus* Essential Oil as New Food Packaging Materials

**DOI:** 10.3390/foods12112169

**Published:** 2023-05-27

**Authors:** Catarina Santos, Ana Ramos, Ângelo Luís, Maria E. Amaral

**Affiliations:** 1 CICS-UBI, Health Sciences Research Centre, University of Beira Interior, Av. Infante D. Henrique, 6200-506 Covilhã, Portugal; catarina.filipa.santos@ubi.pt; 2 FibEnTech-UBI, Fiber Materials and Environmental Technologies Research Unit, University of Beira Interior, Rua Marquês d’Ávila e Bolama, 6201-001 Covilhã, Portugal; ammr@ubi.pt (A.R.); mecca@ubi.pt (M.E.A.); 3 Chemistry Department, Faculty of Sciences, University of Beira Interior, Rua Marquês d’Ávila e Bolama, 6201-001 Covilhã, Portugal

**Keywords:** k-carrageenan, *Cymbopogon winterianus*, essential oil, films, food packaging, antibacterial activity

## Abstract

The global production of synthetic plastics from petroleum-based raw ingredients exceeds 150 million metric tons. The environment is threatened by tons of plastic waste, thus endangering wildlife and the public’s health. These consequences increased the interest in biodegradable polymers as potential substitutes for traditional packaging materials. This study aimed to produce and characterize k-carrageenan films incorporating *Cymbopogon winterianus* essential oil, in which citronellal was determined to be the major compound (41.12%). This essential oil presented remarkable antioxidant activity, as measured through DPPH (IC_50_ = 0.06 ± 0.01%, *v*/*v*; AAI = 85.60 ± 13.42) and β-carotene bleaching (IC_50_ = 3.16 ± 0.48%, *v*/*v*) methods. The essential oil also showed antibacterial properties against *Listeria monocytogenes* LMG 16779 (diameter of inhibition zone = 31.67 ± 5.16 mm and MIC = 8 µL/mL), which were also observed when incorporated in the k-carrageenan films. Moreover, scanning electron microscopy showed the reduction of the biofilms of this bacterium, and even its inactivation, due to visible destruction and loss of integrity when the biofilms were created directly on the developed k-carrageenan films. This study also revealed the quorum sensing inhibition potential of *Cymbopogon winterianus* essential oil (diameter of violacein production inhibition = 10.93 ± 0.81 mm), where it could impede intercellular communication and, hence, lower violacein synthesis. The produced k-carrageenan films were transparent (>90%) and slightly hydrophobic (water contact angle > 90°). This work demonstrated the viability of using *Cymbopogon winterianus* essential oil to produce k-carrageenan bioactive films as new food packaging materials. Future work should focus on the scale-up production of these films.

## 1. Introduction

Plastic-based pollutants have had a significant negative impact on Earth, severely exposing all biotic and abiotic components. Polyurethane (PUR), polystyrene (PS), polyamines (PA), polyvinyl chloride (PVC), poly(-caprolactone) (PCL), polyethylene terephthalate (PET), and many other types of plastics are used in daily life. Due to their advantageous physicochemical properties, they have been widely employed for several applications, namely, packaging, commodities, and hygiene goods [1,2].

Each year, 19 to 23 million tons of plastic waste are dumped into the environment. Recycling plastic presents challenges, especially for single-use plastics. A third of the world’s pollution deposits come from it, which harms the environment, costs a lot to clean, and frequently ends in landfills. In addition, plastic degrades into microplastics, which can reach the food chain and are extremely toxic to all animals [3,4,5,6].

Food needs to be safeguarded against several environmental factors between production and consumption because all foods decay during storage. The properties of the packaging materials must be sufficiently permanent to ensure that the foods’ shelf life is not affected. Plastic packaging, which is most of the plastics market, is responsible for around half of the plastic waste produced globally [7,8,9].

Biodegradable polymers have attracted much attention as potential substitutes for petroleum-based packaging. Several species of linear sulphated polysaccharides, known as carrageenan, which are found in the *Rhodophyceae* family of red seaweeds, have been exploited to create innovative food packaging [10,11]. The principal constituent in cell-wall structures of seaweeds and exoskeletons of crustaceans are marine polysaccharides. A family of polysaccharides made entirely of *D*-galactopyranose units is collectively referred to as carrageenan. They are members of the 3,6-anhydro-*D*-galactose and *D*-galactose family of hydrophilic linear sulphated galactans [12].

Kappa (k), Lambda (λ), Mu (μ), Iota (ι), Theta (θ), and Nu (ν) carrageenans are the most important types. This nomenclature can benefit their chemical classification and commercial manufacturing [13,14,15]. Carrageenan can dissolve in water, thus producing viscous solutions, and are stable to pH variations, due to their unique characteristics. Therefore, they are used in several industrial operations, with the EU additive E-number E407 or E407a when used in food products [12].

There are 140 species in the genus *Cymbopogon* (*Poaceae*), which are more common in Africa, India, Australia, America, Asia, and Europe [16]. While *Cymbopogon winterianus* (*C. winterianus*) (Java citronella) and *Cymbopogon nardus* (Jamarosa) present identical odor and therapeutic applications, they have differing citronellal amounts. Geraniol (36.0%) and citronellal (42.7%) are abundant in java citronella [17].

Although *C. winterianus* essential oil has several applications in aromatherapy, its natural insect-repelling properties make it so well-known. Among other essential oils, citronella oil proved to be the most effective at repelling insects [18]. Regarding therapeutic applications, most citronella essential oils are restricted to their use as a mosquito repellent, antiparasitic, nematocidal, antifungal, and antibacterial agent [19].

This study aimed to evaluate the antioxidant and antibacterial activities of the *C. winterianus* essential oil and incorporate it in k-carrageenan films as new food packaging materials. The major innovation of this work was the use of *C. winterianus* essential oil as a potential food preservative when incorporated in k-carrageenan films, which, to the best of our knowledge, has yet to be exploited. The films were produced through the solvent casting technique, and their barrier, resistance, and bioactive properties were studied.

## 2. Materials and Methods

### 2.1. Biopolymer and Essential Oil

Tokyo Chemical Industry Co., Ltd. (Tokyo, Japan) supplied the k-carrageenan. The *C. winterianus* essential oil was extracted from the plant leaves, which grow spontaneously in Alentejo farmland (Herdade de Vale Côvo, Portugal) (organic farming, PT-BIO-02, ECOCERT). A stainless-steel alembic was used to obtain the essential oil by steam distillation.

### 2.2. Essential Oil Chemical Analysis: Gas Chromatography-Mass Spectrometry (GC-MS)

The *C. winterianus* essential oil chemical composition was studied through Gas Chromatography coupled to Mass Spectrometry (GC-MS) (ISO 7609:1985). The program ran at a temperature of 190 °C for 6 min, before increasing to 190 °C by 2 °C/min, and then to 220 °C by 4 °C/min. After remaining for 10 min at 220 °C, the program switched to its final two modes, which were 4 °C/min up to 240 °C, then staying at that temperature for 10 min. The carrier gas was helium, with injection volumes of 0.1 µL to the Flame Ionization Detector (FID) and 0.1 µL to the Mass Selective Detector (MSD) at head pressures of 33 Psi (FID) and 25.5 Psi (MSD) [20,21].

### 2.3. Antioxidant Activity Evaluation

The *C. winterianus* essential oil antioxidant activity was evaluated using 2,2-diphenyl-1-picrylhydrazyl (DPPH) and β-carotene bleaching methods.

For the DPPH method, 100 µL of several concentrations of the essential oil (5, 3.75, 2.5, 1.25, 0.5, and 0.25%, *v*/*v*) were added to 3.9 mL of three DPPH methanolic solutions (0.2, 0.1242 and 0.08 mM). A mixture of 3.9 mL of each DPPH solution and 100 µL of methanol was used as a negative control. Then, these mixtures remained in the dark at room temperature for 90 min, and their absorbances were measured at 517 nm. The essential oil antioxidant activity was determined as the percentage of inhibition (% Inhibition). Additionally, the IC_50_ was calculated by plotting the essential oil concentrations versus their % Inhibition. The Antioxidant Activity Index (AAI) was calculated, thus allowing the classification of essential oil antioxidant activity (AAI ≤ 0.5—poor; 0.5 < AAI ≤ 1.0—moderate; 1.0 < AAI < 2.0—strong; AAI ≥ 2.0—very strong). The assays were performed in duplicate [22].

For the β-carotene bleaching method, 500 µL of a β-carotene solution (20 mg/mL in chloroform), 1 mL of chloroform, 40 µL of linoleic acid, and 400 µL of Tween 40 were mixed. Then, the chloroform was evaporated with a rotavapor. Finally, 100 mL of distilled water saturated with oxygen was added, thus creating an emulsion. Subsequently, 5 mL of this emulsion was mixed with 300 µL of several essential oil dilutions, as mentioned above, for the DPPH method. The negative control consisted of a mixture of 300 µL of methanol with 5 mL of the emulsion. These mixtures were incubated at 50 °C for 1 h, and, finally, their absorbances were measured at 470 nm. An emulsion without β-carotene was used as a blank. The essential oil antioxidant activity was determined as % Inhibition of β-carotene oxidation, and its IC_50_ was also determined [23].

### 2.4. Antibacterial and Anti-Quorum Sensing Activities Evaluation

The antibacterial activity of the *C. winterianus* essential oil was evaluated against several bacterial species: Gram-negative (*Salmonella* Typhimurium ATCC 13311, *Pseudomonas aeruginosa* ATCC 27853, and *Escherichia coli* ATCC 25922), and Gram-positive (*Staphylococcus aureus* ATCC 25923, *Enterococcus faecalis* ATCC 29212, and *Listeria monocytogenes* LMG 16779). Stock cultures of bacterial species were stored at −80 °C using 20% (*v*/*v*) glycerol. Brain-Heart Infusion agar (BHI) was used to culture the bacteria 24 h prior to the antibacterial tests [23].

For the solid diffusion assay, several bacterial colonies were removed from the agar plates and resuspended in a sterile saline solution (NaCl, 0.85% *w*/*v*). Its turbidity was adjusted to 0.5 McFarland (1–2 × 10^8^ colony-forming units/mL (CFU/mL)). Müeller-Hinton agar (MHA) plates were seeded with these inoculums. An amount of 15 µL of the essential oil was impregnated in sterile cellulose discs (6 mm) and then placed in inoculated Petri dishes. After incubating for 24 h at 37 °C, the inhibition zones were checked and measured using a digital pachymeter [23].

The resazurin microtiter assay was employed to determine the essential oil Minimum Inhibitory Concentrations (MIC). Using Müeller-Hinton broth (MHB), serial two-fold essential oil dilutions (32, 16, 8, 4, 2, 1, 0.5, and 0.25 µL/mL) were made in a 96-well plate (50 µL/well). The resazurin indicator solution (10 µL, 0.1% *w*/*v*) was added to each well, and, finally, 30 µL of MHB was also added. After that, 10 µL of the bacterial suspensions (0.5 McFarland) were added to the wells. The plates were made in triplicate and incubated at 37 °C for 24 h. A visual review allowed us to understand that the color change from purple to pink or colorless was positive. The lowest concentration at which the color shift was observed was found to be the MIC value [23].

Using the biomonitor bacterium, *Chromobacterium violaceum* ATCC 12472, the anti-quorum sensing activity of *C. winterianus* essential oil was evaluated. Luria-Bertani (LB) agar plates were inoculated using a bacterial suspension adjusted to an Optical Density (OD_620nm_) of 1 after the bacterial aerobic growth at 30 °C using LB broth. Then, 15 µL of essential oil was impregnated in sterile cellulose discs (6 mm), which were then placed on the inoculated Petri dishes. After incubating for 24 h at 30 °C, the inhibition of the violacein pigment production around the disc (a ring of colorless, but living, cells) was checked and measured using a digital pachymeter [23,24].

### 2.5. Production of Bioactive Films

The solvent casting technique was used to produce the bioactive films. Initially, 1 g of k-carrageenan was added to 50 mL of distilled water, and this mixture was maintained at 50 °C for 10 min under magnetic stirring to complete the dissolution of the k-carrageenan. To further investigate the effect of incorporating the *C. winterianus* essential oil, 3 different volumes (62.5 µL, 125 µL, and 250 µL) were added to the previous mixture, which was stirred again at 50 °C for 7 min. Finally, 250 µL of glycerol (plasticizer) was added, and the mixture was stirred again under the same conditions. Control films were also prepared without adding the essential oil. Approximately 15 g of the filmogenic solution was placed in polystyrene Petri dishes, and the solvent was left to evaporate for 6 h at 40 °C. Finally, the films were peeled off from the plates and stored at standard room conditions (temperature = 23 ± 2 °C and relative humidity (RH) = 50 ± 5%) [23].

### 2.6. Film Characterization

#### 2.6.1. Fourier-Transform Infrared (FTIR) Spectroscopy

Fourier-Transform Infrared (FTIR) spectra of the bioactive films were acquired between 4000 and 600 cm^−1^, using 64 scans and a resolution of 4 cm^−1^ [25].

#### 2.6.2. Physical Properties

The grammage was determined using the mass-to-area ratio (g/m^2^) (ISO 536:1995). The thickness (µm) was measured with a digital micrometer, taking several random readings (ISO 534:2011) [26].

Tensile strength (N/m), tensile index (N.m/g), peak elongation (%), and elastic modulus (MPa) were determined using a tensile tester, with the initial gap set at 50 mm and with a constant rate of elongation of 10 mm/min (ISO 1924/2) [26].

The optical properties (transparency and color) were evaluated using a Color Touch 2 model ISO spectrophotometer with the following condition: illuminant D65 and observer angle of 10°. The CIE L*a*b* color space enabled the accurate measurements of the films’ color through the evaluation of the lightness (L*), redness/greenness (a*), and yellowness/blueness (b*) coordinates. These measurements were made on several randomly selected points of the films [26].

#### 2.6.3. Contact Angles and Surface Free Energy

The contact angles were measured using three reference liquids (deionized water, ethyleneglycol, and diiodomethane) by applying the sessile drop method. The surface free energy of the films (total, dispersive, and polar components) was calculated with the equipment software that provides the surface tension components of the used liquids. At least six measurements were considered, and the surface free energies of the films were determined through the Owens, Wendt, Rabel, and Kaelble (OWRK) approach [23].

#### 2.6.4. Barrier Properties

Water Vapor Permeability (WVP) (g/Pa.day.m) and Water Vapor Transmission Rate (WVTR) (g/m^2^.day) were analyzed by placing the films on the top of cups containing 15 g of anhydrous CaCl_2_ (desiccant). During 48 h, the weight increase was checked every 2 h, and the test cups were kept at standard room conditions, as mentioned above (ASTM E96-00) [23].

The samples were cut and fixed on the upper part of test tubes, in which 5 mL of edible sunflower seeds oil were added. Then, they were positioned upside-down above a cellulosic filter paper, previously dried (24 h, 105 °C) and weighed to evaluate the Oil Permeability (OP) (g.mm/m^2^ day). The thickness of the films, the filter paper weight increase, the storage time (24 h), and the effective contact area were used to calculate the OP [27].

#### 2.6.5. Antioxidant Activity

The methods described above for *C. winterianus* essential oil were used to quantify the antioxidant activity of the films, with minor changes.

Adding 2.9 mL of a 0.1 mM DPPH solution to 3 discs of the films (6 mm) allowed the measurement of the absorbances at 517 nm every 30 min over 5 h against methanol as a blank. A mixture of the DPPH solution (2.9 mL) and 100 μL methanol was used as a control [27].

By using the β-carotene bleaching method described above for the essential oil, the lipid peroxidation inhibition capacity of the films was evaluated, and the sample was replaced with 3 discs of the films (6 mm) [27].

#### 2.6.6. Antibacterial and Anti-Quorum Sensing Activities

The films’ antibacterial and anti-quorum sensing activities were assessed using the solid diffusion method, outlined above for *C. winterianus* essential oil. Shortly, discs of the films (6 mm) were cut in an aseptic environment and placed on the surface of inoculated Petri dishes [23]. They were also monitored using an optical microscope to verify the bacterial growth inhibition after the incubation period [27]. The results were achieved through three independent assays.

#### 2.6.7. Anti-Biofilm Properties

The anti-biofilm capacity of the films against *Listeria monocytogenes* LMG 16,779 was assessed. For that purpose, the bacterium was grown overnight in Tryptic Soy Broth (TSB) at 250 rpm and 37 °C. Then, the suspension’s turbidity was adjusted to an OD_610nm_ of 0.7, and 300 μL of this suspension was placed on discs of the films (1 cm^2^) that were put on 12-well plates, followed by the addition of 700 μL of TSB. After incubating at 37 °C for 24 h, the biofilms were washed twice with a sterile saline solution and fixed with 2.5% (*v*/*v*) glutaraldehyde for 4 h at 4 °C. Afterward, samples were rewashed with a Phosphate Buffered Saline (PBS) solution and then dehydrated, using a sequence of increasing ethanol concentration solutions. Finally, the samples were dried overnight in a desiccator. The samples were spray-coated with gold, using a metal evaporator, and then were observed through Scanning Electron Microscopy (SEM) (voltage of 20.0 kV and 120.0 A emission) [22,23].

### 2.7. Statistical Analysis

The results were shown as mean ± standard deviation (SD). The raw data were analyzed using the Student’s *t*-test (Microsoft Excel^®^, One Microsoft Way, Redmond, WA, USA), assuming that the continuous variables had a normal distribution. Significant differences were taken if the *p*-value was 0.05 or lower (95% confidence level).

## 3. Results and Discussion

### 3.1. Chemical Composition of C. winterianus Essential Oil

The GC-MS analysis of the essential oil allowed to identify 73 compounds, which correspond to more than 98% of its chemical composition (Table 1). Citronellal, a monoterpenic aldehyde, was identified as the compound present in the highest amount, representing 41.12%, followed by geraniol (19.97%), citronellol (11.94%), along with limonene, geranyl acetate, citronellyl acetate, and elemol, with relative percentages of 3.50%, 2.63%, 2.0%, 1.98%, respectively (Table 1). These results are in accordance with what was previously reported about essential oil-bearing grasses of the genus *Cymbopogon*, focused on *Cymbopogon nardus* [19,22,28,29,30,31].

Citronellal is present in several plants, including those of the *Cymbopogon* genus. It has considerable value in the perfume, cosmetic, and aromatherapy sectors and is the component that gives the essential oil a lemony scent [19]. Citronellal and citronellol are employed as intermediates in synthesizing 1-menthol, α-tocopherol, and irones, which are important products in the fragrance industry [32]. Citronellal has many therapeutic benefits, including antifungal, anthelmintic, anticancer, and antioxidant effects [33,34].

Monoterpenes and sesquiterpenes predominate among the identified compounds (Table 1). The most prevalent types of terpenes are called monoterpenes [35]. They are a vast and diversified class of naturally occurring compounds, many of which are present in essential oils because of their low molecular weight. Several monoterpenes have medicinal benefits that include antimicrobial, anti-inflammatory, antioxidant, antipruritic, and analgesic effects [36]. Sesquiterpenes have a richer aroma, are more stereochemically diverse, are less volatile than terpenes, and have antibacterial and anti-inflammatory properties [37,38].

### 3.2. Antioxidant Activity of the Essential Oil

All foods decay while in storage, since they are perishable, even at varying rates [9]. The reactions that occur during lipid oxidation can produce off-odors, off-flavors, as well as texture and color changes, which affect the consumer’s acceptance and choice [39]. The DPPH method, based on the reduction of reactions and the β-carotene bleaching test, linked to lipid peroxidation, stand out among the known in vitro techniques for assessing the antioxidant capacities of natural products. Thus, these two methods were applied in the evaluation of the antioxidant activity of the essential oil (Table 2) [40].

*C. winterianus* essential oil exhibits important properties, such as antiseptic, anti-inflammatory, and antioxidant [29]. Regarding the results obtained through the DPPH assay, the essential oil displayed a solid antioxidant activity, which is consistent with the previous reports [30]. Moreover, citronellal, the major compound of the essential oil, is a monoterpenic aldehyde, known to possess antioxidant activity. Monoterpenoids, the most abundant compounds in the essential oil, are also recognized, due to their antioxidant properties [36].

One of the determining elements for use in food packaging is the capacity to decrease lipid oxidation, and it is clear from the results of the IC_50_ that very small amounts of *C. winterianus* essential oil are required to inhibit lipid oxidation by 50%. Therefore, it can be concluded that this essential oil can inhibit lipid peroxidation, which can also be related to the high amount of citronellal found in the essential oil (41.12%) and the other monoterpenoids present [36].

To sustainably contribute to the food chain, research has expanded in combining essential oils into packaging produced from materials derived from renewable and biodegradable sources. By having this promising result, the *C. winterianus* essential oil has the potential to be used in active food packaging, whose organoleptic properties are highly dependent on the preservation of the lipid content. [41].

### 3.3. Antibacterial and Anti-Quorum Sensing Properties of the Essential Oil

Concerning the antibacterial activity of the *C. winterianus* essential oil, Gram-positive bacteria, particularly *Listeria monocytogenes* LMG 16779, were more susceptible than Gram-negative ones (Table 3). By determining their MIC values (Table 3), the antibacterial activity of the essential oil was further assessed. The lowest MIC values were found for Gram-positive bacteria, specifically for *Listeria monocytogenes* LMG 16,779 and *Enterococcus faecalis* ATCC 29212, thus supporting the findings from the solid diffusion assay. Gram-positive bacteria are known to be more susceptible than Gram-negative ones, since they do not have an external membrane [42].

The opportunistic pathogen *Listeria monocytogenes* leads to life-threatening infections in both humans and animals. This facultative intracellular bacterium is common in nature, and it contaminates a wide variety of surfaces through cross-contamination during the development of biofilms in industrial machinery, plumbing, and other surfaces, thus reaching the final product for consumption [43]. Eating foods that have been contaminated can result in illnesses [44]. Therefore, in the context of food packaging, the activity that the *C. winterianus* essential oil demonstrated against *Listeria monocytogenes* LMG 16,779 is interesting because it would inhibit its growth.

In a sort of microbial communication, called quorum sensing, bacteria communicate and work together through various chemical signals, called auto-inducers, to keep a certain process in balance and impact populations. Additionally, it affects how genes are expressed in response to cell density, and triggers the activation of several transcription factors in bacteria [45]. This essential oil was able to inhibit the violacein production by *Chromobacterium violaceum* ATCC 12,472 (Table 3); thus, it was possible to confirm its anti-quorum sensing capacity.

### 3.4. FTIR Spectra of the Films

**Figure 1:**
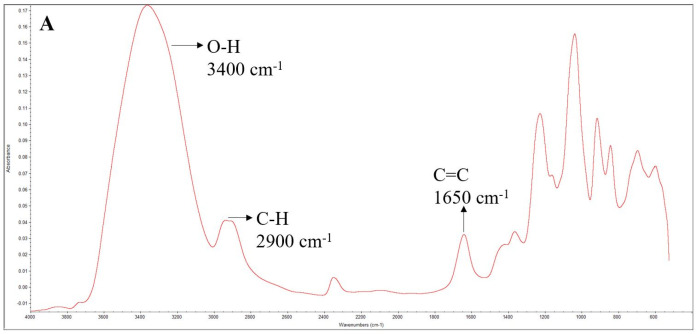

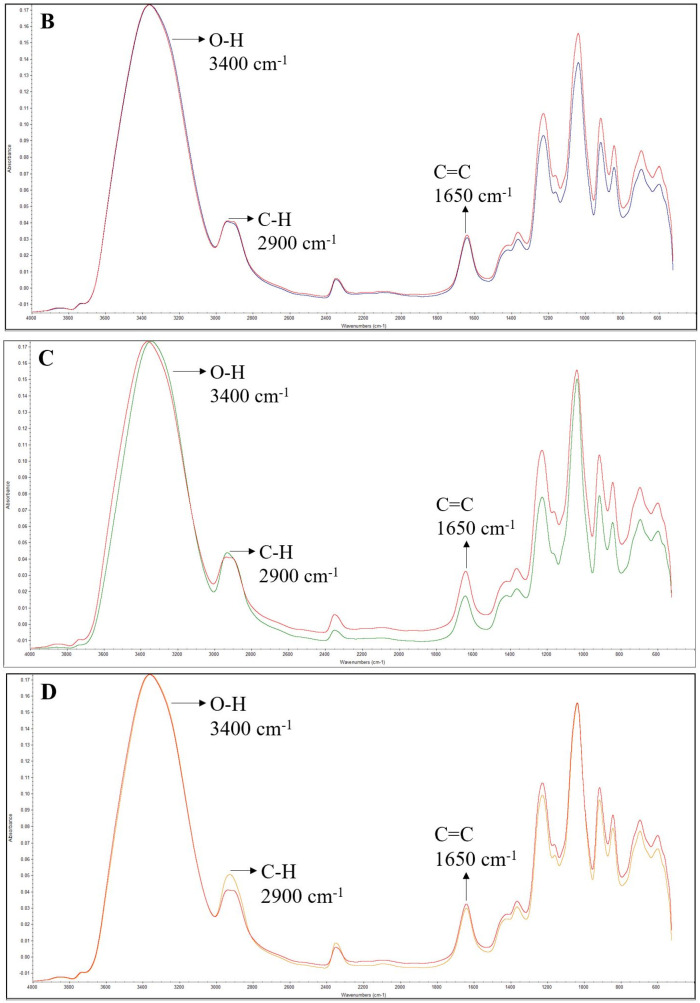
FTIR spectra of the control film (**A**); incorporating 62.5 µL (**B**), 125 µL (**C**), and 250 µL (**D**) of *C. winterianus* essential oil.

The results of the FTIR analysis of the films are shown in Figure 1.

The FTIR spectrum of the k-carrageenan film (Figure 1A) showed several representative bands: at 3000–3500 cm^−1^, corresponding to O-H stretching, and at 1220–845 cm^−1^, known as the fingerprint region of carbohydrates [46]. The peak at 1220 cm^−1^ corresponds to the asymmetric stretching of ester sulfate groups (O=S=O), 1030 cm^−1^ is assigned to C-O and C-OH stretching, and 930 cm^−1^ corresponds to C-O-C stretching in 3,6-anhydro-*D*-galactose. The band at 845 cm^−1^ is related to C-O-SO_3_ stretching in (1-3)-*D*-galactose. The intense bands at 1592 cm^−1^ and 1382 cm^−1^ are probably related to the structural water deformation band [46].

The fingerprint area in FTIR spectra is in the range between 400 cm^−1^ and 1500 cm^−1^. It typically has many peaks, thus making it challenging to distinguish between them and pick out individual bonds. However, since each compound’s fingerprint region is distinct, it is possible to distinguish one compound from another [47].

The FTIR spectra of the k-carrageenan films incorporating the essential oil (Figure 1B–D) showed that the existing peaks were higher, even though no new peaks have appeared, thus indicating that the essential oil compounds were present as their concentration increased. The observed peaks corresponded to the O-H bond at 3400 cm^−1^, the C-H bond at 2900 cm^−1^, and the C=C bond at 1650 cm^−1^. Generally, a difference in the peaks’ intensity was noticed. As the volume of the essential oil incorporated in the films increased, their bands also increased in the FTIR spectra, thus showing that the essential oil was present in the films. Since the concentration of the *C. winterianus* essential oil is much lower than that of k-carrageenan, the peaks corresponding to citronellal (the major compound) are superimposed with those of k-carrageenan, as shown in Figure 1.

### 3.5. Physical Properties of the Films

Table 4 shows the effects of incorporating the essential oil on the films’ structural, mechanical, and optical properties. The k-carrageenan films’ thickness varied from 38.51 to 64.93 mm, with the incorporation of the *C. winterianus* essential oil significantly affecting (*p*-value < 0.05) the thickness of the resulting films, as well as the grammage, due to the difference in the total solid contents of the films [26]. Visually, it was feasible to confirm that the roughness increases as the amount of essential oil in the films increases. The surface roughness may increase because of the essential oils’ incorporation into the polymer matrix, thus affecting their thickness [48].

Tensile strength, tensile index, peak elongation, and elastic modulus were evaluated as the films’ main mechanical properties that depend on the type of the polymer matrix, the sort of additives, and the interactions between them [49]. The ability to endure a tensile force is measured by assessing the breakpoint of a specimen under tensile stress [50].

Increasing the content of the essential oil in the films causes a reduction in tensile strength and tensile index, which significantly changed (*p*-value < 0.05) when the volume was 250 mL, but did not change significantly (*p*-value > 0.05) for the other essential oil volumes. In the film network, weaker polymer-essential oil connections have partially replaced stronger polymer-polymer interactions, which is a possible reason for the reduction [51,52]. Peak elongation is the increase in the specimen’s length, from its starting to its breakpoint length, which is related to the elasticity of a polymeric material. In this study, peak elongation significantly increased (*p*-value < 0.05) as the volume of essential oil also increased to 62.5 µL and 125 µL. The peak elongation obtained for the film with 250 µL of *C. winterianus* essential oil was lower than that of the control, thus suggesting the existence of fewer interconnections within the film structure, thus limiting its flexibility. As previously observed, the elastic modulus decreased with the increase of the essential oil content incorporated in the films, hence resulting in less rigid films [53]. Adding the essential oil to the polymer may not result in a homogenous combination, giving the films a roughness that creates minuscule weak areas and makes them more prone to breaking [48,49]. When combining polysaccharides and essential oils, the mechanical properties are frequently reduced [50,51,54].

The mechanical properties of the k-carrageenan films now developed are weaker when compared with plastic films used in the food industry, such as low-density polyethylene (LDPE) [55].

Since color and transparency directly affect the consumers’ acceptance, the optical properties of the films were evaluated (Table 4). Adding *C. winterianus* essential oil did not significantly modify (*p*-value > 0.05) the L*, a*, and b* coordinates. The incorporation of essential oil decreased the transparency values; nonetheless, the films showed high transparency values, between 92% and 96%, which might be related to the amorphous arrangement in k-carrageenan films and connected to the light scattering brought on by the distribution of the essential oil droplets inside the biopolymer matrix [56]. This conclusion is consistent with findings from other studies, since adding more essential oil often makes film less transparent [53,54].

### 3.6. Contact Angle and Surface Free Energies

The most popular technique for determining the hydrophobicity or hydrophilicity of a surface is to measure the water contact angle. A hydrophobic surface has a water contact angle superior to 90°, whereas a hydrophilic one has a water contact angle inferior to 90° [57]. The values of the contact angles were obtained on both sides of the films and are listed in Table 5. It was crucial to carefully examine both sides of the films, because they displayed extremely different behaviors. This can be important when deciding which side to pack food on. If it is desirable to pack food with more water content, then the hydrophobic side must be turned inward to prevent water loss.

Generally, there were no changes in the hydrophobicity of the films regarding the upper surface. Moreover, the upper surface is hydrophobic, contrary to the lower one, which has a contact angle inferior to 90°, and this tends to decrease with the increase of essential oil volumes. It was also noticed that the water contact angles obtained in the control films were similar for both sides, whereas this was not the case with the addition of the essential oil. Looking at the films with essential oil, a decrease in hydrophobicity on the bottom surfaces was observed, while, on the upper ones, the hydrophobicity tends to increase. Essential oils are hydrophobic, due to their non-polar chemical structure, which is the reason why incorporating them into hydrophilic polymer matrices increases the barrier properties [52]. In addition to the essential oil’s natural hydrophobicity, another explanation for the rise in water contact angle may be related to the roughness of the films, which may also increase their hydrophobicity, since the surface of the structures is crucial in determining a surface’s hydrophobicity [58].

There are numerous applications for surface free energy, which establishes how solids will perform in the presence of liquids. High surface free energy materials are often easily wettable by any liquid [59]. Measurements of contact angles were frequently used to calculate the material’s surface free energy for some liquids [60]. The total surface free energy of the films varied from 13.95 to 38.60 mN/m, and, analyzing its evolution, along with the increase of *C. winterianus* essential oil, it increased relatively to the control film. The polar component decreased with the addition of the essential oil, with no statistical significance (*p*-value > 0.05). The dispersive element also tended to increase with the addition of the essential oil. The total surface free energy values obtained from the films were comparable to some petroleum-derived plastics, such as polypropylene [61].

### 3.7. Barrier Properties

Food products should be transported, handled, and commercialized in a protective environment, created by food packaging materials. By serving as an effective barrier to moisture and gases, they should help extend the shelf life of perishable foods [62].

The barrier properties of the films developed in this work were evaluated in terms of barrier-to-water vapor and oil (Table 6). Regarding the water vapor permeability of the films, it was observed that adding the essential oil did not significantly change (*p*-value > 0.05) the WVTR and WVP of the k-carrageenan films. These results are aligned with the values obtained for the water contact angles of the films, which indicated that the essential oil did not affect the hydrophobicity of the films, and suggests that the barrier properties to water vapor will also not be affected.

Considering the oil permeability results, they lacked a clear pattern and were somewhat dispersed; however, they did not show statistically significant results (*p*-value > 0.05). A previous study with k-carrageenan films reported values of oil permeability of 0.37 and 0.97 g.mm/m^2^.day, which are lower than those obtained in the present research [63].

It has been previously established that water retention in polysaccharide-based films is connected to potential interactions between polysaccharide molecules and glycerol hydroxyl groups, thus resulting in a more compact polymeric matrix [64].

### 3.8. Antioxidant Activity of the Films

**Figure 2:**
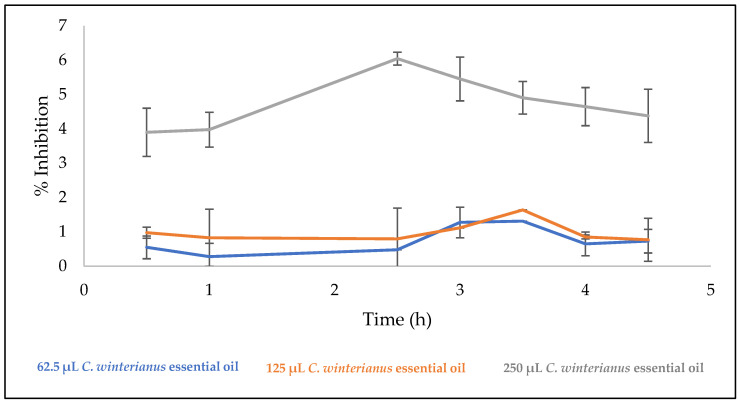
Antioxidant activity of the films evaluated by DPPH radical. Results are presented as mean ± SD.

The results of the films’ antioxidant activity determined by the DPPH assay are shown in Figure 2. The control film did not show antioxidant activity, which does not happen with the films incorporating essential oil. An antioxidant activity pattern was observed for films containing the essential oil. After the initial phase, they remained steady, but after 2 h, an increase in the antioxidant activity was verified. After 2.5 h of reaction, the films with 250 µL of essential oil showed 6%, and, at 4 h, they showed 5% of DPPH free radicals inhibition. The films containing 62.5 and 125 µL of essential oil showed only one major peak: almost 1.5% and 2.8% inhibition of DPPH free radicals, respectively.

The results of the β-carotene bleaching test, in which the ability of the films to inhibit lipid peroxidation was evaluated, are presented in Table 7. The findings demonstrated that films containing the essential oil exhibit a high level of lipid peroxidation inhibition, and, for that reason, they have the potential to be used as substitutes for traditional food packaging materials, particularly for foods with high-fat content.

### 3.9. Antibacterial and Anti-Quorum Sensing Properties of the Films

**Figure 3:**
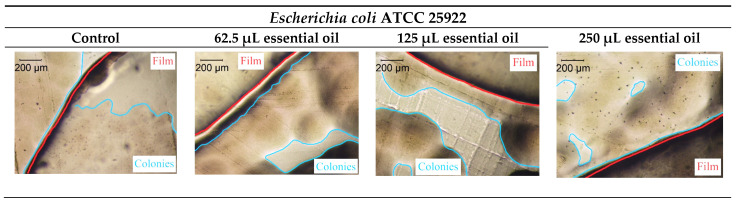

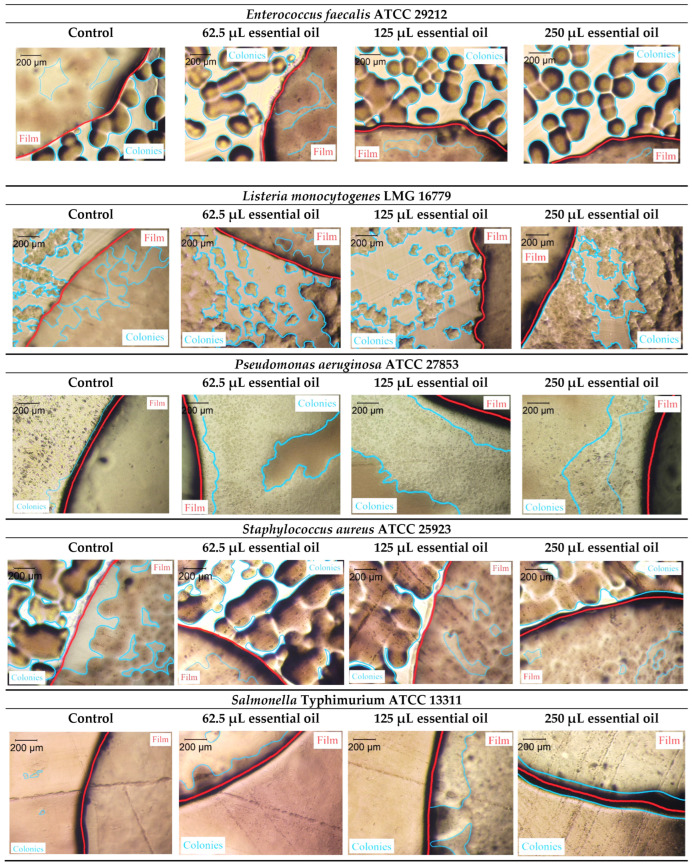
Optical microscopy images of the antibacterial activity of the films (scale bar = 200 µm).

The solid diffusion method was used to assess the films’ antibacterial and anti-quorum sensing properties and analyze the inhibition zones (Table 8). It was possible to verify that, in some cases, there was contact inhibition of bacterial growth (6 mm), for example, in 125 μL and 250 μL in *Listeria monocytogenes* LMG 16,779 and the control film with the bacterium *Salmonella* Typhimurium ATCC 13311. The antibacterial activity results were also verified through optical microscopy, and the colonies and the edge of the film were marked in blue and red, respectively. Comparing the findings of the values of Table 8 with the pictures in Figure 3, it can be concluded that, for the strains that did not exhibit inhibition, bacterial growth takes place below the film.

Regarding the anti-quorum properties of the films, it was noticed that the essential oil capacity to inhibit bacterial intercellular communication was not maintained when it was incorporated in k-carrageenan films.

### 3.10. Anti-Biofilm Activity of Film against Listeria monocytogenes

**Figure 4:**
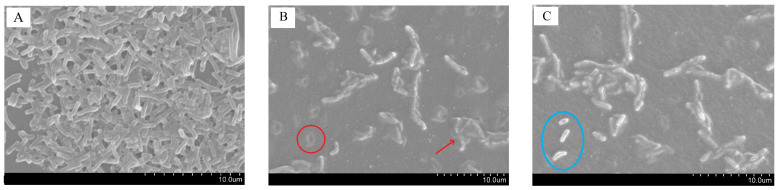
SEM images of *Listeria monocytogenes* LGM 16,779 biofilms formed directly on the surface of the control film (**A**) and on the film containing 250 µL of *C. winterianus* essential oil (**B**,**C**) (scale bar = 10 µm, red lines show destroyed bacterial cells; blue circle show intact bacterial cells).

Since the *C. winterianus* essential oil primarily affected the growth of *L. monocytogenes* LMG 16779, and because *Listeria monocytogenes* is a well-known foodborne pathogen, the antibiofilm activity of the films incorporating 250 µL of essential oil were evaluated through SEM (Figure 4). These films were chosen because the increase in the volume of essential oil incorporated in the films would present a better antibiofilm effect, since the essential oil has an antibacterial activity for this bacterium [65,66].

Figure 4A presents the *Listeria monocytogenes* biofilm formed in the control film, showing several layers of bacteria. As shown in Figure 4B, biofilms formed on the surface of k-carrageenan films containing 250 µL of essential oil are sparse and consist of just one layer of cells. In addition, it was also visible that the cell integrity was lost (examples marked in red). Figure 4C shows that the number of bacteria decreased when the biofilms were formed on the bioactive k-carrageenan films. Some bacteria have compromised cell membrane integrity, but there were also some bacteria with good integrity for comparison (examples marked in blue).

## 4. Conclusions

This work demonstrated the viability of using *C. winterianus* essential oil to produce k-carrageenan bioactive and eco-friendly films that can be used as innovative food packaging materials, thus avoiding using conventional plastics. The produced films were transparent (>90%) and hydrophobic (water contact angle > 90°), and presented antioxidant activity related to free radical scavenging and lipid peroxidation inhibition. Moreover, the films inhibited the growth of planktonic and biofilm cells of *Listeria monocytogenes,* an important foodborne pathogen. Future work should focus on industrial production at a large scale of these films and study their biodegradability under different conditions.

## Figures and Tables

**Table 1:** Chemical composition of the *C. winterianus* essential oil.

Retention Time (min)	Compounds	% Relative	Chemical Family
6.09	Acetone	0.02	Aliphatic ketone
13.06	Tricyclene	0.01	Monoterpene
13.85	α-Pinene	0.22	Monoterpene
16.49	Camphene	0.04	Monoterpene
19.18	β-Pinene	0.01	Monoterpene
20.04	Sabinene	0.01	Monoterpene
21.99	Δ-3-Carene	0.02	Monoterpene
22.89	β-Myrcene	0.06	Monoterpene
23.13	α-Phellandrene	0.01	Monoterpene
25.58	Limonene	3.50	Monoterpene
26.29	β-Phellandrene	0.02	Monoterpene
26.60	1,8-Cineole	0.07	Monoterpenic ether
28.02	*cis*-β-Ocimene	0.11	Monoterpene
28.97	γ-Terpinene	0.01	Monoterpene
29.26	*trans*-β-Ocimene	0.06	Monoterpene
30.69	*p*-Cymene	0.02	Monoterpene
31.72	α-Terpinolene	0.05	Monoterpene
35.51	6-Methyl-5-Hepten-2-one	0.07	Aliphatic ketone
36.77	Melonal	0.08	Aliphatic aldehyde
36.99	*cis*-Rose oxide	0.02	Monoterpenic ether
38.13	*trans*-Rose oxide	0.01	Monoterpenic ether
44.62	α-Cubebene	0.01	Sesquiterpene
45.08	Menthone	0.04	Monoterpenic ketone
45.79	Citronellal	41.12	Monoterpenic aldehyde
48.98	β-Bourbonene	0.08	Sesquiterpene
50.00	Linalool	0.75	Monoterpenic alcohol
51.71	neo-Isopulegol	0.42	Monoterpenic alcohol
52.21	Isopulegol	1.25	Monoterpenic alcohol
53.11	*trans*-α-Bergamotene	0.07	Sesquiterpene
53.45	β-Elemene	1.17	Sesquiterpene
53.87	β-Copaene	0.04	Sesquiterpene
54.11	Terpinen-4-ol	0.02	Monoterpenic alcohol
54.24	*trans*-β-Caryophyllene	0.31	Sesquiterpene
54.91	Citronellyl formate	0.04	Monoterpenic ester
55.75	*cis*-β-Terpineol	0.05	Monoterpenic alcohol
56.43	*trans*-Muurola-3,5-diene	0.02	Sesquiterpene
57.63	Citronellyl acetate	2.00	Monoterpenic ester
58.09	*trans*-Cadina-1(6),4-diene	0.03	Sesquiterpene
58.89	α-Humulene	0.08	Sesquiterpene
58.98	Neral	0.63	Monoterpenic aldehyde
59.88	α-Amorphene	0.21	Sesquiterpene
60.17	Germacrene-*D*	1.09	Sesquiterpene
61.49	(*Z*,*E*)-α-Farnesene	0.12	Sesquiterpene
62.02	Geranial	1.28	Monoterpenic aldehyde
62.76	Bicyclogermacrene	0.01	Sesquiterpene
63.37	Geranyl acetate	2.63	Monoterpenic ester
63.83	Citronellol	11.94	Monoterpenic alcohol
63.93	Δ-Cadinene	1.21	Sesquiterpene
64.25	γ-Cadinene	0.51	Sesquiterpene
65.01	Lavandul	0.01	Monoterpenic alcohol
65.59	*trans*-Cadina-1,4-diene	0.04	Sesquiterpene
65.86	Nerol	0.19	Monoterpenic alcohol
66.14	α-Cadinene	0.12	Sesquiterpene
68.54	Geraniol	19.97	Monoterpenic alcohol
71.48	Geranyl butyrate	0.15	Monoterpenic alcohol
71.71	*cis*-Muurol-5-en-4-β-ol	0.11	Sesquiterpenic alcohol
74.59	*trans*-Muurol-5-en-4-β-ol	0.13	Sesquiterpenic alcohol
77.48	Methyl eugenol	0.06	Phenylpropanoid
80.15	Germacrene-*D*-4-ol	1.11	Sesquiterpenic alcohol
80.63	1,10-Di-Epi-Cubenol	0.07	Sesquiterpenic alcohol
81.03	1-Epi-Cubenol	0.02	Sesquiterpenic alcohol
81.24	Elemol	1.98	Sesquiterpenic alcohol
81.93	8-Hidroxy-neo-menthol	0.18	Alcohol
84.28	Eugenol	0.90	Phenylpropanoid
84.83	*trans*-Methyl-isoeugenol	0.16	Alcohol
85.79	*T*-Muurolol	0.25	Sesquiterpenic alcohol
86.23	α-Muurolol	0.07	Sesquiterpenic alcohol
86.70	Elemicine	0.05	Ether
87.30	Citronellic acid	0.26	Carboxylic acid
87.42	α-Eudesmol	0.16	Sesquiterpenic alcohol
87.75	α-Cadinol	0.55	Sesquiterpenic alcohol
87.87	β-Eudesmol	0.15	Sesquiterpenic alcohol
92.87	(*E*,*E)*-Farnesol	0.06	Sesquiterpenic alcohol
Cumulative amount		43.03	Monoterpenic aldehydes
		34.75	Monoterpenic alcohols
		5.12	Sesquiterpenes
		4.67	Monoterpenic esters
		4.66	Sesquiterpenic alcohols
		4.15	Monoterpenes
		0.96	Phenylpropanoids
		0.34	Alcohols
		0.26	Carboxylic acids
		0.10	Monoterpenic ethers
		0.09	Aliphatic ketones
		0.08	Aliphatic aldehydes
		0.05	Ethers
		0.04	Monoterpenic ketones

**Table 2:** Antioxidant properties of the *C. winterianus* essential oil.

Method	Parameters	*C. winterianus* Essential Oil
DPPH	IC_50_ (%, *v*/*v*)	0.06 ± 0.01
	AAI	85.60 ± 13.42
	Antioxidant activity	Very strong
β-Carotene bleaching	IC_50_ (%, *v*/*v*)	3.16 ± 0.48

Results are presented as mean ± SD.

**Table 3:** Antibacterial and anti-quorum sensing properties of the *C. winterianus* essential oil.

Strains	Diameter of Inhibition Zones (mm) ^1^	MIC Values (µL/mL) ^2^
*S. aureus* ATCC 25923	15.53 ± 2.12	32
*L. monocytogenes* LMG 16779	31.67 ± 5.16	8
*E. faecalis* ATCC 29212	12.03 ± 0.18	16
*E. coli* ATCC 25922	9.98 ± 0.66	>32
*S.* Typhimurium ATCC 13311	6.00 ± 0.00	32
*P. aeruginosa* ATCC 27853	7.77 ± 0.00	>32
*C. violaceum* ATCC 12472	10.93 ± 0.81	-

^1^ Results are presented as mean ± SD; ^2^ Results are presented as modal values.

**Table 4:** Physical properties of the films.

Properties		Control ^a^	62.5 µL Essential Oil ^b^	125 µL Essential Oil ^c^	250 µL Essential Oil ^d^	*p*-Values
Structural	Grammage (g/m^2^)	65.18 ± 2.91	65.36 ± 5.26	66.43 ± 5.87	69.12 ± 4.09	0.926 ^ab^ 0.557 ^ac^ 0.030 ^ad^*
	Thickness (µm)	38.51 ± 4.01	41.45 ± 4.98	44.17 ± 5.51	64.93 ±7.89	0.002 ^ab^* <0.001 ^ac^* <0.001 ^ad^*
Mechanical	Tensile strength (N/m)	3006.99 ± 80.56	2783.54 ± 236.82	2821.52 ± 201.98	1432.29 ± 14,38	0.242 ^ab^ 0.256 ^ac^ 0.019 ^ad^*
	Tensile index (N.m/g)	46.15 ± 1.20	42.60 ± 3.63	42.46 ± 3.02	20.75 ± 0.21	0.230 ^ab^ 0.163 ^ac^ 0.018 ^ad^*
	Peak elongation (%)	1.91 ± 0.01	2.38 ± 0.09	3.73 ± 0.47	1.46 ± 0.12	0.009 ^ab^* 0.021 ^ac^* 0.114 ^ad^
	Elastic modulus (MPa)	7978.67 ± 312.77	6946.07 ± 545.55	5558.45 ± 337.19	3017.49 ± 2.64	0.075 ^ab^ 0.008 ^ac^* 0.028 ^ad^*
Optical	L* (Lightness)	93.01 ± 0.23	93.15 ± 0.17	93.26 ± 0.15	94.07 ± 0.51	0.448 ^ab^ 0.201 ^ac^ 0.052 ^ad^
	a* (Redness)	1.58 ± 0.04	1.60 ± 0.07	1.51 ± 0.02	1.33 ± 0.08	0.676 ^ab^ 0.069 ^ac^ 0.018 ^ad^*
	b* (Yellowness)	−5.94 ± 0.11	−5.93 ± 0.40	−5.56 ± 0.11	−4.38 ± 0.42	0.970 ^ab^ 0.014 ^ac^* 0.018 ^ad^*
	Transparency (%)	95.72 ± 0.454	94.54 ± 0.384	93.83 ± 0.348	91.65 ± 1.463	0.028 ^ab^* 0.006 ^ac^* 0.031 ^ad^*

Results are presented as mean ± SD; * Indicates a significant result (*p*-value < 0.05); Superscript letters (^a–d^) indicate the samples under statistical comparison.

**Table 5:** Contact angles and surface free energy of the films.

Films		Water Contact Angle (◦)	Diiodomethane Contact Angle (◦)	Ethyleneglycol Contact Angle (◦)	Dispersive Component, ɤ^D^ (mN/m)	Polar Component, ɤ^P^ (mN/m)	Total Surface Free Energy, ɤ^T^ (mN/m)
Control	Top face ^a^	93.22 ± 5.02	93.16 ± 5.58	93.22 ± 5.02	9.84 ± 2.08	4.35 ± 1.88	14.19 ± 2.80
	Bottom face ^b^	93.09 ± 5.60	93.90 ± 4.92	93.22 ± 5.02	9.97 ± 1.87	3.98 ± 1.79	13.95 ± 2.59
62.5 µL essential oil	Top face ^c^	97.95 ± 6.98	47.27 ± 0.96	62.81 ± 2.48	35.79 ± 0.52	0.38 ± 0.25	36.17 ± 0.58
	Bottom face ^d^	93.39 ± 4.79	47.04 ± 1.70	60.00 ± 1.95	35.90 ± 0.92	0.74 ± 0.30	36.64 ± 0.97
125 µL essential oil	Top face ^e^	86.81 ± 8.44	41.67 ± 2.86	66.82 ± 0.84	38.53 ± 1.46	0.00 ± 0.01	38.53 ± 1.46
	Bottom face ^f^	69.00 ± 8.44	41.45 ± 2.83	73.77 ± 5.08	38.40 ± 1.44	0.20 ± 0.37	38.60 ± 1.49
250 µL essential oil	Top face ^g^	95.10 ± 4.76	44.26 ± 4.74	65.50 ± 5.43	37.11 ± 2.46	0.23 ± 0.36	37.35 ± 2.49
	Bottom face ^h^	84.43 ± 5.47	50.47 ± 1.38	79.09 ± 0.75	33.72 ± 0.77	0.33 ± 0.12	34.05 ± 0.78
*p*-values		0.210 ^ac^ 0.933 ^bd^ 0.147 ^ae^ 0.004 ^bf^* 0.495 ^ag^ 0.031 ^bh^*	<0.001 ^ac^* <0.001 ^bd^* <0.001 ^ae^* <0.001 ^bf^* <0.001 ^ag^* <0.001 ^bh^*	<0.001 ^ac^* <0.001 ^bd^* <0.001 ^ae^* <0.001 ^bf^* <0.001 ^ag^* <0.001 ^bh^*	<0.001 ^ac^* <0.001 ^bd^* <0.001 ^ae^* <0.001 ^bf^* <0.001 ^ag^* <0.001 ^bh^*	0.065 ^ac^ 0.085 ^bd^ 0.057 ^ae^ 0.062 ^bf^ 0.058 ^ag^ 0.071 ^bh^	<0.001 ^ac^* <0.001 ^bd^* <0.001 ^ae^* <0.001 ^bf^* <0.001 ^ag^* 0.003 ^bh^*

Results are presented as mean ± SD; * Indicates a significant result (*p*-value < 0.05); Superscript letters (from ^a^ to ^h^) indicate the samples under statistical comparison.

**Table 6:** Barrier properties of the films.

Films	Water Vapor		Oil
	WVTR (g/m^2^.day)	WVP (g/Pa.day.m) (×10^−5^)	(g.mm/m^2^.day)
Control ^a^	532.58 ± 1.76	1.55 ± 0.01	4.79 ± 0.39
62.5 μL essential oil ^b^	534.14 ± 33.95	1.68 ± 0.11	13.32 ± 2.65
125 μL essential oil ^c^	503.27 ± 12.35	1.68 ± 0.04	6.93 ± 1.06
250 μL essential oil ^d^	539.75 ± 8.38	2.65 ± 0.04	9.64 ± 2.08
*p*-values	0.959 ^ab^ 0.178 ^ac^ 0.434 ^ad^	0.348 ^ab^ 0.136 ^ac^ 0.015 ^ad^*	0.401 ^ab^ 0.127 ^ac^ 0.509 ^ad^

Results are presented as mean ± SD; * Indicates a significant result (*p*-value < 0.05); Superscript letters (^a–d^) indicate the samples under statistical comparison.

**Table 7:** Antioxidant properties of the films determined by β-carotene bleaching test.

Films	% Inhibition	*p*-Values
Control ^a^	79.35 ± 5.64	-
62.5 μL essential oil ^b^	90.14 ± 2.68	0.375 ^ab^
125 μL essential oil ^c^	100.00 ± 3.29	0.068 ^ac^
250 μL essential oil ^d^	100.00 ± 4.64	0.355 ^ad^

Results are presented as mean ± SD; Superscript letters (^a–d^) indicate the samples under statistical comparison.

**Table 8:** Diameters of the inhibition zones (mm).

Strains	Films			
	Control	62.5 μL Essential Oil	125 μL Essential Oil	250 μL Essential Oil
*S. aureus* ATCC 25923	0.00 ± 0.00	0.00 ± 0.00	0.00 ± 0.00	0.00 ± 0.00
*L. monocytogenes* LMG 16779	0.00 ± 0.00	0.00 ± 0.00	6.00 ± 0.00	6.00 ± 0.00
*E. faecalis* ATCC 29212	0.00 ± 0.00	0.00 ± 0.00	0.00 ± 0.00	0.00 ± 0.00
*E. coli* ATCC 25922	0.00 ± 0.00	0.00 ± 0.00	0.00 ± 0.00	0.00 ± 0.00
*S.* Typhimurium ATCC 13311	6.00 ± 0.00	0.00 ± 0.00	0.00 ± 0.00	0.00 ± 0.00
*P. aeruginosa* ATCC 27853	6.85 ± 1.20	7.93 ± 0.67	8.17 ± 0.37	7.62 ± 0.16
*C. violaceum* ATCC 12472	6.00 ± 0.00	6.00 ± 0.00	6.00 ± 0.00	6.00 ± 0.00

Results are presented as mean ± SD.

## Data Availability

Data will be available by request to the corresponding author.

## References

[1] Abou-Zeid D.M., Müller R.J., Deckwer W.D. (2001). Degradation of natural and synthetic polyesters under anaerobic conditions. J. Biotechnol..

[2] Vroman I., Tighzert L. (2009). Biodegradable polymers. Materials.

[3] Borrelle S.B., Ringma J., Law K.L., Monnahan C.C., Lebreton L., McGivern A., Murphy E., Jambeck J., Leonard G.H., Rochman C.M. (2020). Mitigate Plastic Pollution. Science.

[4] Brandon J.A., Jones W., Ohman M.D. (2019). Multidecadal increase in plastic particles in coastal ocean sediments. Sci. Adv..

[5] Lim X.Z. (2021). Microplastics are everywhere-but are they harmful?. Nature.

[6] Delangiz N., Aliyar S., Pashapoor N., Nobaharan K., Lajayer B.A., Rodríguez-Couto S. (2022). Can polymer-degrading microorganisms solve the bottleneck of plastics’ environmental challenges?. Chemosphere.

[7] Xie Q., Liu G., Zhang Y., Yu J., Wang Y., Ma X. (2022). Active edible films with plant extracts: A updated review of their types, preparations, reinforcing properties, and applications in muscle foods packaging and preservation. Crit. Rev. Food Sci. Nutr..

[8] Xu J., Yang Z., Wang Z., Li J., Zhang X. (2023). Flexible sensing enabled packaging performance optimization system (FS-PPOS) for lamb loss reduction control in E-commerce supply chain. Food Control.

[9] Man C.M.D. (2016). Food Storage Trials.

[10] Ansar B.S.K., Kavusi E., Dehghanian Z., Pandey J., Lajayer B.A., Price G.W., Astatkie T. (2022). Removal of organic and inorganic contaminants from the air, soil, and water by algae. Environ. Sci. Pollut. Res..

[11] Zarina S., Ahmad I. (2015). Biodegradable composite films based on k-carrageenan reinforced by cellulose nanocrystal from kenaf fibers. Bioresources.

[12] Pereira L. (2016). Carrageenans: Sources and Extraction Methods, Molecular Structure, Bioactive Properties and Health Effects.

[13] Necas J., Bartosikova L. (2013). Carrageenan: A review. Vet. Med..

[14] Zia K.M., Tabasum S.T., Nasif M., Sultan N., Aslam N., Noreen A., Zuber M. (2017). A review on synthesis, properties and applications of natural polyme- based carrageenan blends and composites. Int. J. Biol. Macromol..

[15] Campo V.L., Kawano D.F., Silva D.B., Carvalho I. (2009). Carrageenans: Biological properties, chemical modifications and structural analysis—A review. Carbohydr. Polym..

[16] Fatima S., Abad A.H.F., Sharma S. (2002). Physiological and metabolic responses of different genotypes of Cymbopogon martinii and C. winterianus to water stress. Plant Growth Regul..

[17] Blank A.F., Costa A.G., Arrigoni-Blank M.F., Cavalcanti S.C.H., Alvez P.B., Innecco R., Ehlert P.A.D., Sousa I.F. (2007). Influence of season, harvest time and drying on Java citronella (*Cymbopogon winterianus* Jowitt) volatile oil. Bras. Farmacogn..

[18] Curtis C.F., Lines J.D., Ijumba J., Callaghan A., Hill N. (1987). The relative efficacy of repellents against mosquito vectors of disease. Med. Vet. Etomol..

[19] Wany A., Jha S., Nigam V.K., Pandey D.M. (2013). Chemical analysis and therapeutic uses of citronella oil from Cymbopogon winterianus: A short review. Int. J. Adv. Res..

[20] Sedikelo G.K., Lenetha G.G., Malebo N.J. (2022). Chromatography-mass spectrometry and chemical characteristics of Thymus zygis and Cymbopogon winterianus essential oils: Possible insect repellents. Sci. Afr..

[21] Verma R.S., Verma S.K., Tandon S., Padalia R.C., Darokar M.P. (2020). Chemical composition and antimicrobial activity of Java citronella (*Cymbopogon winterianus* Jowitt ex Bor) essential oil extracted by different methods. J. Essent. Oil Res..

[22] Luís Â., Pereira L., Domingues F., Ramos A. (2019). Development of a carboxymethyl xylan film containing licorice essential oil with antioxidant properties to inhibit the growth of foodborne pathogens. LWT Food Sci. Technol..

[23] Luís Â., Ramos A., Domingues F. (2020). Pullulan films containing rockrose essential oil for potential food packaging applications. Antibiotics.

[24] Kamath A., Shukla A., Patel D. (2023). Quorum Sensing and Quorum Quenching: Two sides of the same coin. Physiol. Mol. Plant Pathol..

[25] Luís Â., Domingues F., Ramos A. (2019). Production of hydrophobic zein-based films bioinspired by the lotus leaf surface: Characterization and bioactive properties. Microorganisms.

[26] Luís Â., Gallardo E., Ramos A., Domingues F. (2020). Design and characterization of bioactive bilayer films: Release kinetics of isopropyl palmitate. Antibiotics.

[27] Luís Â., Ramos A., Domingues F. (2021). Pullulan–apple fiber biocomposite films: Optical, mechanical, barrier, antioxidant and antibacterial properties. Polymers.

[28] Luthra R., Singh N., Sharma S. (1991). Changes in monoterpene content accompanying development of Cymbopogon winterianus Jowitt leaves. J. Essent. Oil Res..

[29] Rodrigues K.A.F., Dias C.N., Amaral F.M.M.A., Moraes D.F.C., Filho V.E.M., Andrade E.H.A., Maia J.G.S. (2013). Molluscicidal and larvicidal activities and essential oil composition of *Cymbopogon winterianus*. Pharm. Biol..

[30] Leite B.L.S., Bonfim R.R., Antoniolli A.R., Thomazzi S.M., Araújo A.A.S., Blank A.F., Estevam C.S., Cambui E.V.F., Bonjardim L.R., Júnior R.L.C.A. (2010). Assessment of antinociceptive, anti-inflammatory and antioxidant properties of Cymbopogon winterianus leaf essential oil. Pharm. Biol..

[31] Kaur H., Bhardwaj U., Kaur R. (2021). Cymbopogon nardus essential oil: A comprehensive review on its chemistry and bioactivity. J. Essent. Oil Res..

[32] Victoria F.N., Radatz C.S., Sachini M., Jacob R.G., Alves D., Savegnago L., Perin G., Motta A.S., Silva W.P., Lenardão E.J. (2012). Further analysis of the antimicrobial activity of α-phenylseleno citronellal and α-phenylseleno citronellol. Food Control.

[33] Fatima K., Luqman S. (2021). Citronellal suppress the activity of ornithine decarboxylase in hypopharyngeal carcinoma cells. S. Afr. J. Bot..

[34] Nakahara K., Alzoreky N.S., Yoshihashi T., Nguyen H.T.T., Trakoontivakorn G. (2013). Chemical Composition and Antifungal Activity of Essential Oil from Cymbopogon nardus (Citronella Grass). Jpn. Agric. Res. Q..

[35] Xie S., Wu G., Ren R., Xie R., Yin H., Chen H., Yang B., Zhang Z., Maosheng G. (2023). Transcriptomic and metabolic analyses reveal differences in monoterpene profiles and the underlying molecular mechanisms in six grape varieties with different flavors. LWT Food Sci. Technol..

[36] Zielińska-Błajet M., Feder-Kubis J. (2020). Monoterpenes and their derivatives—Recent development in biological and medical applications. Int. J. Mol. Sci..

[37] Waterman P.G. (1993). Volatile Oil Crops: Their Biology, Biochemistry, and Production.

[38] Ishnava K.B., Chauhan J.B., Barad M.B. (2013). Anticariogenic and phytochemical evaluation of Eucalyptus globules Labill. Saudi J. Biol. Sci..

[39] Domínguez R., Barba F.J., Gómez B., Putnik P., Kovaceviv D.B., Pateiro M., Santos E.M., Lorenzo J.M. (2018). Active packaging films with natural antioxidants to be used in meat industry: A review. Food Res. Int..

[40] Sacchetti G., Maietti S., Muzzoli M., Scaglianti M., Manfredini S., Radice M., Bruni R. (2005). Comparative evaluation of 11 essential oils of different origin as functional antioxidants, antiradicals and antimicrobials in foods. Food Chem..

[41] Ribeiro-Santos R., Andrade M., Melo N.R., Sanches-Silva A. (2017). Use of essential oils in active food packaging: Recent advances and future trends. Trends Food Sci. Technol..

[42] Simic A., Rancic A., Sokovic M.D., Ristic M., Grujic-Jovanovic S., Vukojevic J., Marin P.D. (2008). Essential oil composition of Cymbopogon winterianus and Carum carvi and their antimicrobial activities. Pharm. Biol..

[43] Camargo A.C., Woodward J.J., Call D.R., Nero L.A. (2017). Listeria monocytogenes in Food-Processing Facilities, Food Contamination, and Human Listeriosis: The Brazilian Scenario. Foodborne Pathog. Dis..

[44] Ragon M., Wirth T., Hollandt F., Lavenir R., Lecuit M., Monnier A.L., Brisse S. (2008). A new perspective on Listeria monocytogenes evolution. PLoS Pathog..

[45] Babalola O.O. (2010). Beneficial bacteria of agricultural importance. Biotechnol. Lett..

[46] Gulzar S., Balange A.K., Nagarajarao R.C., Zhao Q., Benjakul S. (2022). Microcapsules of Shrimp Oil Using Kidney Bean Protein Isolate and k-Carrageenan as Wall Materials with the Aid of Ultrasonication or High-Pressure Microfluidization: Characteristics and Oxidative Stability. Foods.

[47] Ramírez-Hernández A., Aguilar-Flores C., Aparicio-Saguilán A. (2019). Fingerprint analysis of FTIR spectra of polymers containing vinyl acetate. DYNA.

[48] Ojagh S.M., Rezaei M., Razavi S.H., Hosseini S.M.H. (2010). Development and evaluation of a novel biodegradable film made from chitosan and cinnamon essential oil with low affinity toward water. Food Chem..

[49] Zhang W., Shu C., Chen Q., Cao J., Jiang W. (2019). The multi-layer film system improved the release and retention properties of cinnamon essential oil and its application as coating in inhibition to penicillium expansion of apple fruit. Food Chem..

[50] Liang J., Yan H., Zhang J., Dai W., Gao X., Zhou Y., Wan X., Puligundla P. (2017). Preparation and characterization of antioxidant edible chitosan films incorporated with epigallocatechin gallate nanocapsules. Carbohydr. Polym..

[51] Sharma S., Barkauskaite S., Jaiswal A.K., Jaiswal S. (2021). Essential oils as additives in active food packaging. Food Chem..

[52] Sánchez-González L., Vargas M., González-Martínez C., Chiralt A., Cháfer M. (2009). Characterization of edible films based on hydroxypropylmethylcellulose and tea tree essential oil. Food Hydrocoll..

[53] Barizão C.L., Crepaldi M.I., Junior O.O.S., Oliveira A.C., Martins A.F., Garcia P.S., Bonafé E.G. (2020). Biodegradable films based on commercial k-carrageenan and cassava starch to achieve low production costs. Int. J. Biol. Macromol..

[54] Atarés L., Chiralt A. (2016). Essential oils as additives in biodegradable films and coatings for active food packaging. Trends Food Sci. Technol..

[55] Szlachetka O., Witkowska-Dobrev J., Baryła A., Dohojda M. (2021). Low-density polyethylene (LDPE) building films–Tensile properties and surface morphology. J. Build. Eng..

[56] Sánchez-González L., Cháfer M., Chiralt A., González-Martínez C. (2010). Physical properties of edible chitosan films containing bergamot essential oil and their inhibitory action on Penicillium italicum. Carbohydr. Polym..

[57] Niu B., Shao P., Chen H., Sun P. (2019). Structural and physiochemical characterization of novel hydrophobic packaging films based on pullulan derivatives for fruits preservation. Carbohydr. Polym..

[58] Carpena M., Nuñez-Estevez B., Soria-Lopez A., Garcia-Oliveira P., Prieto M.A. (2021). Essential Oils and Their Application on Active Packaging Systems: A Review. Resources.

[59] Wang G., Guo Z., Liu W. (2014). Interfacial effects of superhydrophobic plant surfaces: A Review. J. Bionic Eng..

[60] What is Surface Free Energy? Biolin Scientific. https://www.biolinscientific.com/blog/what-is-surface-free-energy.

[61] Rbihi S., Aboulouard A., Laallam L., Jouaiti A. (2020). Contact Angle Measurements of Cellulose based Thin Film composites: Wettability, surface free energy and surface hardness. Surf. Interfaces.

[62] Chibowski E., Terpilowski K. (2009). Surface free energy of polypropylene and polycarbonate solidifying at different solid surfaces. Appl. Surf. Sci..

[63] Sousa A.M.M., Gonçalves M.P. (2015). Strategies to improve the mechanical strength and water resistance of agar films for food packaging applications. Carbohydr. Polym..

[64] Farhan A., Hani N.M. (2017). Characterization of edible packaging films based on semi-refined k-carrageenan plasticized with glycerol and sorbitol. Food Hydrocoll..

[65] Tonyali B., Cikrikci S., Oztop M.H. (2018). Physicochemical and microstructural characterization of gum tragacanth added whey protei- based films. Food Res. Int..

[66] Hadjilouka A., Mavrogiannis G., Mallouchos A., Paramithiotis S., Mataragas M., Drosinos E.H. (2017). Effect of lemongrass essential oil on Listeria monocytogenes gene expression. LWT Food Sci. Technol..

